# Phase 1 study of GSK3368715, a type I PRMT inhibitor, in patients with advanced solid tumors

**DOI:** 10.1038/s41416-023-02276-0

**Published:** 2023-05-26

**Authors:** Anthony B. El-Khoueiry, James Clarke, Tobias Neff, Tim Crossman, Nirav Ratia, Chetan Rathi, Paul Noto, Aarti Tarkar, Ignacio Garrido-Laguna, Emiliano Calvo, Jordi Rodón, Ben Tran, Peter J. O’Dwyer, Adam Cuker, Albiruni R. Abdul Razak

**Affiliations:** 1 University of Southern California Norris Comprehensive Cancer Center, 1441 Eastlake Ave, Los Angeles, CA USA; 2 GSK, Gunnels Wood Road, Stevenage, Hertfordshire SG1 2NY UK; 3 GSK, 1250 S Collegeville Road, Collegeville, PA USA; 4 Huntsman Cancer Institute, 2000 Cir of Hope Dr, Salt Lake City, UT USA; 5 START Madrid-CIOCC, Centro Integral Oncológico Clara Campal, Calle Oña, 10, 28050 Madrid, Spain; 6 Investigational Cancer Therapeutics Department, University of Texas MD Anderson Cancer Center, 1400 Holcombe Blvd Unit 455, 8th Floor, Houston, TX USA; 7 Peter MacCallum Cancer Centre (PMCC), 305 Grattan Street, Melbourne, VIC 3000 Australia; 8 University of Pennsylvania, Abramson Cancer Center, 3400 Civic Center Blvd, Philadelphia, PA USA; 9 Perelman School of Medicine, University of Pennsylvania, 3400 Spruce St, Philadelphia, PA USA; 10 Phase 1 Program, Princess Margaret Cancer Centre, 610 University Ave, Toronto, M5G2M9 ON Canada; 11 Present Address: Merck&Co, North Wales, PA USA; 12 Present Address: Adaptimmune LLC, Philadelphia, PA USA

**Keywords:** Cancer, Targeted therapies

## Abstract

**Background:**

GSK3368715, a first-in-class, reversible inhibitor of type I protein methyltransferases (PRMTs) demonstrated anticancer activity in preclinical studies. This Phase 1 study (NCT03666988) evaluated safety, pharmacokinetics, pharmacodynamics, and preliminary efficacy of GSK3368715 in adults with advanced-stage solid tumors.

**Methods:**

In part 1, escalating doses of oral once-daily GSK3368715 (50, 100, and 200 mg) were evaluated. Enrollment was paused at 200 mg following a higher-than-expected incidence of thromboembolic events (TEEs) among the first 19 participants, resuming under a protocol amendment starting at 100 mg. Part 2 (to evaluate preliminary efficacy) was not initiated.

**Results:**

Dose-limiting toxicities were reported in 3/12 (25%) patients at 200 mg. Nine of 31 (29%) patients across dose groups experienced 12 TEEs (8 grade 3 events and 1 grade 5 pulmonary embolism). Best response achieved was stable disease, occurring in 9/31 (29%) patients. Following single and repeat dosing, GSK3368715 maximum plasma concentration was reached within 1 h post dosing. Target engagement was observed in the blood, but was modest and variable in tumor biopsies at 100 mg.

**Conclusion:**

Based on higher-than-expected incidence of TEEs, limited target engagement at lower doses, and lack of observed clinical efficacy, a risk/benefit analysis led to early study termination.

**Trial registration number:**

NCT03666988.

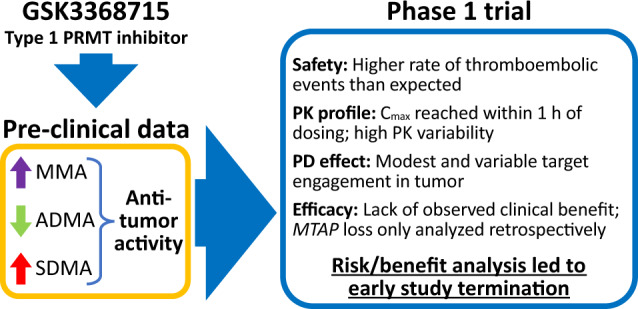

## Background

Arginine methylation is an important posttranslational modification of proteins involved in diverse cellular processes such as gene regulation, ribonucleic acid (RNA) processing, mRNA splicing, deoxyribonucleic acid (DNA) repair, and signal transduction [1–4]. A family of enzymes (types I, II, and III protein methyltransferases [PRMTs]) catalyzes these reactions. Type I PRMTs are primarily responsible for generating asymmetric dimethylarginine (ADMA), type II catalyzes the symmetrically demethylated arginine derivative (SDMA), and type III catalyzes monomethylarginine (MMA). Overexpression of type I PRMTs leads to epigenetic modifications that play a role in regulating gene expression and oncogenesis [5, 6]. Through the methylation of arginine residues on histone and non-histone substrates, type 1 PRMTs contribute to the transformation, proliferation, invasiveness, and survival of tumor cells in a number of solid (bladder, breast, colon, glioblastoma multiforme, kidney, melanoma, non-small cell lung cancer, pancreatic, and prostate) and hematopoietic cancers (acute myeloid leukemia, lymphomas, and myeloma). Disruption of ADMA modification through the inhibition of type I PRMTs may decrease tumor cell proliferation [6].

GSK3368715 is a potent, reversible, S-adenosylmethionine (SAM)-uncompetitive inhibitor that binds to the protein substrate binding pocket of type I PRMTs. Inhibition of type I PRMTs reduces intracellular ADMA and leads to accumulation of MMA and SDMA [7]. In preclinical cancer models, GSK3368715 induced maximal decreases in global ADMA levels after 72 h and strong anti-proliferative activity in multiple tumor types [6]. Cytostatic responses were observed in the majority of solid tumors tested, and deficiency of the enzyme methylthioadenosine phosphorylase (MTAP) in pancreatic cells was associated with a cytotoxic response. Cytotoxic responses were also observed in 56% of lymphomas and 50% of acute myeloid leukemia (AML) cell lines. There is also some evidence that tumors with a high dependency on splicing may be susceptible to further modulation of splicing through type I PRMT inhibition.

Genetic loss of the *MTAP* gene leads to intracellular accumulation of the MTAP metabolite, 2-methylthioadenosine (MTA). This inhibits activity of the type II PRMT enzyme, PRMT5 [6, 8], which has known roles in tumorigenesis. Endogenous inhibition of PRMT5 may also render MTAP-deficient cancers more sensitive to type I PRMT inhibition [8, 9]. Indeed, preclinical studies in mice treated with the combination of GSK3368715 and GSK3326595 (a PRMT5 inhibitor), suggest that inhibition of both type I PRMTs and PRMT5 may have synergistic effects [6]. Inhibition of tumor growth in pancreatic and diffuse large B-cell lymphoma (DLBCL) cell lines was greater with both agents in combination relative to either agent alone. Additionally, since *MTAP* is frequently deleted in human cancers due to its proximity to the tumor suppressor gene *CDKN2A*, the therapeutic potential of type I PRMT inhibition alone merits investigation.

With or without MTAP loss, overexpression of PRMTs may represent a targetable vulnerability in many tumor types [9–11]. Therefore, the primary objective of this first-time-in-human study was to determine the recommended Phase 2 dose for GSK3368715 in participants with selected advanced-stage solid tumors. Additionally, safety, pharmacokinetics (PK), pharmacodynamics (PD), and preliminary clinical efficacy of GSK3368715 were assessed.

## Methods

### Study design

**Fig. 1:**
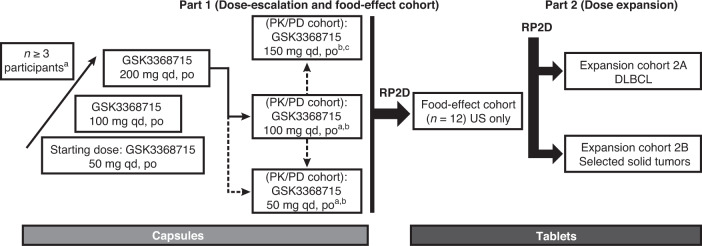
Planned study design. DLBCL diffuse large B-cell lymphoma, PD pharmacodynamic, PK pharmacokinetic, po orally, qd once daily, RP2D recommended Phase 2 dose. ^a^Participants with advanced/refractory solid tumors. ^b^Under protocol amendment 03, enrollment resumed in the 100-mg PK/PD cohort and the 50-mg PK/PD cohort. ^c^Dose escalation was limited to 50-mg increments.

This was a Phase 1, open-label study consisting of a dose escalation, followed by a planned dose-expansion cohort of oral administration of GSK3368715 conducted between October 26, 2018 and March 4, 2021 (Fig. 1). The study was approved by the ethics committee at every participating institution and was conducted according to the recommendations of Good Clinical Practice and the Declaration of Helsinki. All patients provided written informed consent to participate in the study.

Part 1 of this study included a dose-escalation cohort to assess the incidence of dose-limiting toxicities (DLTs) and adverse events, and a PK/PD cohort to characterize the PK/PD profile of GSK3368715 (Fig. 1). A food-effect cohort and a second study phase (part 2) to assess the preliminary clinical activity of GSK3368715 were also planned.

The starting dose for the dose escalation cohort was 50 mg. Based on toxicity studies in dogs and preclinical studies of tumor regression in mice, tumor regression was observed at daily doses ranging from >75 mg/kg to 300 mg/kg (depending on tumor type), and 50 mg provided 16- and 33-fold safety margins for the steady state AUC and *C*_max_, respectively. The Neuenschwander Continual Reassessment Method dose-escalation design was used to identify the next dose level during dose escalation (see ’Statistical analyses’) [12].

For each dosing group in the dose-escalation cohort, GSK3368715 was administered on day 1, no treatment was administered on days 2 and 3 (to characterize single-dose PK), and daily dosing continued thereafter until disease progression, unacceptable toxicity, or withdrawal of consent. Study treatment was dosed at approximately the same time of day (±4 h), with no food for 1 h before and 2 h after each dose. Evaluable patients for dose escalation received at least 21 days of study intervention and completed the postintervention follow-up visit.

Due to a higher-than-expected incidence of thromboembolic events (TEEs) among the first 19 participants in the dose-escalation cohort, enrollment was paused at the 200-mg dose, and several measures were implemented to reduce the risk of TEE in subsequent patients enrolled in the study. The study resumed under a protocol amendment with the PK/PD cohort starting at a daily dose of 100 mg, and dose escalation was limited to 50-mg increments. The incidence and frequency of TEEs were added to the DLT criteria (grade 2 TEE requiring systemic anticoagulation or ≥grade 3 TEE) with extended monitoring to 8 weeks or until study discontinuation, whichever occurred first. Further, eligibility criteria were modified to exclude patients at high risk of thrombosis (ie, Khorana score ≥3 or prior medical history of TEE). Patients with a Khorana score of 2 were considered for prophylactic anticoagulation if deemed appropriate by the investigator. Khorana score is a risk assessment model based on clinical and laboratory parameters to classify the risk for chemotherapy-associated venous thromboembolism (Supplemental Table 2) [13].

The study was halted prior to initiation of part 2 due to a comprehensive risk/benefit analysis. No recommended Phase 2 dose was determined and no food effect analysis was performed as planned in part 1.

### Study population

Full inclusion/exclusion criteria for both parts 1 and 2 are included in Supplemental Table 1. Participants for Phase 1 were ≥18 years of age with histologically- or cytologically confirmed metastatic or nonresectable solid tumors who had exhausted standard treatment options (>1 but not >4 lines of prior therapy). MTAP status was recorded but deficiency was not required. Adequate organ function as defined by hematology and chemistry values and an Eastern cooperative oncology group (ECOG) performance status of 0 or 1 were also required. Participants in the PK/PD cohort for part 1 consented to a biopsy at screening and for one on-treatment biopsy.

As mentioned above, following the study amendment, patients at high risk of venous thromboembolism as defined by either Khorana Score of ≥3, or prior medical history of venous thromboembolism, were ineligible.

### Study assessments

Adverse events were coded using the standard MedDRA groupings and graded according to NCI-CTCAE version 5.0. Clinical chemistry, urinalysis, and coagulation tests were performed predose on days 1, 8, 15, 22, and weekly thereafter. Tumor imaging occurred every 8 weeks until week 33, and then every 16 weeks thereafter.

In both the dose-escalation and PK/PD cohorts, plasma samples for PK analysis were obtained following dosing on day 1 and pre- and postdose on days 2, 3, 4, 8, 15, 16, 22, and predose every 4 weeks thereafter. Plasma concentrations for GSK3368715 and its metabolites (GSK3963583, GSK3983164, and GSK3510519) were quantified using a validated ultra high-performance liquid chromatography-mass spectrometry-mass spectrometry (LC-MS-MS) method. PK parameters were analyzed using noncompartmental methods.

MTAP loss was determined by IHC using formalin-fixed paraffin. A positive result was defined by a complete loss (absence of IHC cytoplasmic staining) in tumor cells or partial loss (reduced cytoplasmic staining or heterogeneous staining). A negative result was defined by retained staining (no loss of cytoplasmic staining) of tumor cells as compared with the retained staining of the internal control lymphocytes. MTAP status was distinctly binary where the cytoplasmic expression for a whole tumor sample was determined to be either lost (positive) or retained (negative).

Tumor cell and plasma target engagement PD biomarkers were assessed in the dose-escalation cohort on days 1, 8, 15, and 22. In the PK/PD cohort, urine for PK and metabolite profiling was collected postdose on day 1 and through 48 h postdose on day 2, and for 24 h postdose on day 15. Participants underwent an on-treatment tumor biopsy on day 15. Asymmetric arginine 225 (R225) methylation of heterogeneous nuclear ribonucleoprotein A1 (hnRNP-A1) was identified as a target engagement PD of type I PRMT inhibition; treatment with GSK3368715 results in the reduction of asymmetric dimethylarginine 225 (ADMA-R225) on hnRNP-A1 protein in cancer cell lines and peripheral blood mononuclear cells (PBMCs). Noto et al. describe in detail the identification of hnRNP-A1 as a pharmacodynamic biomarker of type I PRMT inhibition, and the development of novel methodologies to accurately and precisely quantitate changes in the levels of ADMA on hnRNP-A1 in both blood and tumor compartments [14]. Levels of ADMA-R225-hnRNP-A1 were measured in fresh frozen and formalin-fixed paired biopsies by liquid chromatography-mass spectrometry (LC-MS) and immunohistochemistry (IHC).

To measure the ADMA-R225-hnRNP-A1 in the PBMCs, blood was collected using the standard technique for BD Vacutainer® Evacuated Blood Collection Tubes. The PBMC aspirate prepared was used for the ADMA analyses as previously described by the LC-MS method [14].

### Statistical analyses

The Neuenschwander Continual Reassessment Method dose-escalation design has been previously described. Briefly, the dose level with the highest posterior probability of having a DLT rate within the target toxicity range (≥16% and <33%) was recommended for the next cohort. Additionally, following the protocol amendment, dose escalations were limited to 50-mg increments.

Adverse events and DLTs were summarized descriptively by dose cohort. Pharmacokinetic parameters of GSK3368715 and its metabolites (GSK3963583 and GSK3983164) were estimated in the PK/PD population (all participants for whom a PK sample was obtained) using a noncompartmental analysis model (WinNonlin version 8.1) and summarized descriptively. Best overall response as per RECIST 1.1 was summarized descriptively by dose cohort. Levels of tumor cell and plasma pharmacodynamic biomarkers were also summarized descriptively.

## Results

### Patient disposition and baseline characteristics

A total of 31 patients were enrolled and received treatment (50 mg, *n* = 3; 100 mg, *n* = 16; 200 mg, *n* = 12). Patient mean (SD) age was 58.3 (13.23) years. Over two-thirds were female, most were white, and a variety of tumor types were included (Table 1). Six patients (19%) had MTAP gene loss (100 mg, 2 patients; 200 mg, 4 patients).

**Table 1:** Patient demographics and clinical characteristics.

Patient characteristic	Safety population			Total *N* = 31
	50 mg *n* = 3	100 mg *n* = 16	200 mg *n* = 12	
Age, years				
Mean (SD)	68.0 (6.6)	55.5 (15.1)	59.7 (10.9)	58.3 (13.2)
Sex, *n* (%)				
Male	2 (67)	4 (25)	3 (25)	9 (29)
Female	1 (33)	12 (75)	9 (75)	22 (71)
Ethnicity				
Not Hispanic or Latino	3 (100)	15 (94)	9 (75)	27 (87)
Hispanic or Latino	0	1 (6)	3 (25)	4 (13)
Race, *n* (%)^a^				
White	3 (100)	14 (88)	10 (83)	27 (87)
Asian	0	2 (13)	1 (9)	3 (10)
Primary tumor type, *n* (%)				
Colon/rectum	0	2 (13)	4 (33)	6 (19)
Pancreas	1 (33)	2 (13)	1 (8)	4 (13)
Ovary	1 (33)	0	1 (8)	2 (6)
Bladder	0	0	1 (8)	1 (3)
Breast	0	1 (6)	0	1 (3)
Cervix	0	1 (6)	0	1 (3)
Cholangiocarcinoma in gallbladder	0	1 (6)	0	1 (3)
Desmoplastic small round cell tumor	0	1 (6)	0	1 (3)
Epithelioid hemangioendothelioma	0	0 (0)	1 (8)	1 (3)
Gastric	0	1 (6)	0	1 (3)
Head and neck	0	1 (6)	0	1 (3)
Liver	1 (33)	0 (0)	0	1 (3)
Malignant neoplasm of parotid gland	0	1 (6)	0	1 (3)
Malignant perivascular epithelioid cell neoplasm	0	1 (6)	0	1 (3)
Melanoma	0	1 (6)	0	1 (3)
Mucoepidermoid cancer of parotid	0	0 (0)	1 (8)	1 (3)
Nasopharynx carcinoma	0	1 (6)	0 (0)	1 (3)
Non-small cell lung	0	0 (0)	1 (8)	1 (3)
Osteosarcoma	0	1 (6)	0	1 (3)
Pleomorphic adenoma	0	1 (6)	0 (0)	1 (3)
Prostate	0	0	1 (8)	1 (3)
Uterine perivascular epithelioid cell tumor	0	0	1 (8)	1 (3)
MTAP loss, *n* (%)	–	2 (13)	4 (33)	6 (19)
Time since diagnosis, days Median (min, max)	735 (685, 1259)	596 (359, 3487)	923 (321, 2898)	765 (321, 3487)

*Max* maximum, *min* minimum, *MTAP* methylthioadenosine phosphorylase, *SD* standard deviation.

^a^Race was not available for one patient in the 200-mg dose group.

When the study was initially paused, there were 3 patients receiving the 50 mg, 4 receiving 100 mg, and 12 in the 200-mg dose group. When the study was halted early, 20 patients were evaluable, having completed the 21-day DLT window.

The majority of patients (23 [74%]) discontinued treatment due to disease progression, 5 patients discontinued due to an AE, and 3 discontinued due to a DLT. Nineteen (61%) patients died during the study due to disease progression. One patient with pancreatic cancer and a recent history of pulmonary embolism (PE) had a fatal PE.

### Safety

Three (25%) patients had DLTs (aortic thrombosis, atrial fibrillation, and decrease in platelet count) in the 200-mg dose group, which are discussed further below. There were no DLTs reported in the 50 mg or 100-mg dose groups.

Nearly all (30 [97%]) patients experienced ≥1 treatment emergent AE (Table 2), with nausea (9 [29%]), anemia (9 [29%]), and fatigue (8 [26%]) reported most frequently overall. Sixteen (52%) patients had ≥1 grade 3 or grade 4 treatment emergent AEs. In the 100-mg dose group, a grade 3 decrease in neutrophil count led to a dose reduction for 1 patient. In the 200-mg dose group, grade 3 deep vein thrombosis, aortic thrombosis, and atrial fibrillation were reported in 1 patient each, 2 patients experienced a grade 4 decrease in platelet count, and 1 patient experienced a grade 4 decrease in lymphocyte count.

**Table 2:** Safety and adverse events.

Preferred term	GSK3368715 dose			
	50 mg (*n* = 3)	100 mg (*n* = 16)	200 mg (*n* = 12)	Total (*N* = 31)
Any AE^a^	3 (100)	15 (94)	12 (100)	30 (97)
>10% of participants, *n* (%)				
Nausea	2 (67)	3 (19)	4 (33)	9 (29)
Anemia	1 (33)	6 (38)^b^	2 (17)	9 (29)
Fatigue	1 (33)	6 (38)	1 (8)	8 (26)
Diarrhea	1 (33)	5 (31)^b^	1 (8)	7 (23)
Vomiting	2 (67)	2 (13)	3 (25)	7 (23)
Pyrexia	1 (33)	5 (31)	0	6 (19)
Pulmonary embolism	0	2 (13)^c^	3 (25)^f^	5 (16)
Neutrophil count decreased	0	1 (6)^d,e^	3 (25)	4 (13)
Dyspnea	1 (33)	2 (13)	1 (8)	4 (13)
GSK3368715-related AEs by maximum grade, *n* (%)				
Grade 3				4 (13)
Aortic thrombosis	0	0	1 (8)^b,g^	
Deep vein thrombosis	0	0	1 (8)	
Atrial fibrillation	0	0	1 (8)^g^	
Neutrophil count decreased	0	1 (6)^e^	0	
Grade 4				2 (6)
Platelet count decreased	0	0	1 (8)^b,g^	
Lymphocyte count decreased	0	0	1 (8)^b^	

*AE* adverse event.

^a^Includes all AEs and all grades.

^b^Led to dose interruption in 1 participant.

^c^Led to permanent discontinuation in 1 participant.

^d^Possibly study drug related.

^e^Led to a dose reduction.

^f^Fatal in 1 participant.

^g^Categorized as dose-limiting toxicity.

Twelve (39%) patients had ≥1 serious AE (SAE), with 2 fatal (grade 5) SAEs in the 200-mg dose group (PE in 1 patient with a history of prior PE and intracranial hemorrhage in 1 patient with brain metastases).

No clinically significant trends were observed in changes from baseline for clinical chemistry, hematology, urinalysis, or echocardiograms (ECGs).

#### Thromboembolic events

A total of 9 (29%) participants experienced 12 TEEs (Table 3), only 1 of which occurred after the study resumed following the protocol amendment. In the 50-mg dose group, 1 participant had a grade 2 portal vein thrombosis. In the 100-mg dose group, 2 pulmonary embolisms were reported (1 was grade 2 and 1 was grade 3). In the 200-mg dose group, 8 grade 3 events were reported (4 pulmonary embolism events, 2 portal vein thrombosis events, 1 deep vein thrombosis, and 1 aortic thrombosis). Additionally, as noted above, 1 participant receiving 200 mg experienced a grade 5 pulmonary embolism. This patient had pancreatic cancer and a recent history of venous thromboembolism and was receiving low molecular weight heparin. Both patients in the 100-mg dose group and 3 of those who experienced a TEE in the 200-mg dose group had Khorana scores [13] <3 at study entry and no prior history of TEE. The treatment of TEEs was as per institutional guidelines and consistent with accepted clinical practice, ie, initially with heparin/low molecular weight heparin, followed by oral anticoagulation (Table 3). Following the protocol amendment, prophylactic anticoagulation was permitted in patients with a Khorana score of 2 if investigators felt it was appropriate. Three of the 12 patients enrolled after the amendment received prophylactic anticoagulation (oral acetylsalicylic acid 100 mg once daily, oral enoxaparin 40 mg once daily, and subcutaneous enoxaparin 40 mg once daily); none of these patients experienced a TEE.

**Table 3:** Thromboembolic events.

GSK3368715 dose^a^	Primary neoplasm at diagnosis	Thromboembolic event	Grade	Anticoagulation status	
				Started prior to study treatment	Stated medication(s)
50 mg	Cholangiocarcinoma	Portal vein thrombosis	2	Not known	Not known
100 mg	Pancreas adenocarcinoma	Pulmonary embolism	3	No	Enoxaparin 40 mg QD intramuscularly
	Head and neck squamous cell carcinoma	Pulmonary embolism^b^	2	No	Bemiparin 7500 units intradermally
200 mg	Pancreas adenocarcinoma mucinous	Pulmonary embolism^c^	5	No	Heparin 25,000 units continuous infusion Enoxaparin sodium 60 mg BID subcutaneously Rivaroxaban 15 mg QD oral
	Colon/rectum adenocarcinoma	Portal vein thrombosis Pulmonary embolism	3 3	No	Enoxaparin sodium 60 mg BID subcutaneously
	NSCLC adenocarcinoma	Aortic thrombosis^d,e^ Portal vein thrombosis	3 3	No	Enoxaparin sodium 60 mg BID subcutaneously
	Mucoepidermoid carcinoma of parotid	Pulmonary embolism	3	No	Enoxaparin sodium 60 mg BID subcutaneously edoxaban 60 mg QD oral
	Epithelioid hemangioendothelioma	Pulmonary embolism	3	No	Enoxaparin sodium 60 mg BID subcutaneously Rivaroxaban 15 mg QD oral
	Prostate adenocarcinoma	Deep vein thrombosis^d,e^ Pulmonary embolism	3 3	No	Enoxaparin sodium QD subcutaneously Rivaroxaban 15 mg QD oral

*BID* twice daily, *QD* once daily.

^a^*n* = 24.

^b^Study drug withdrawn.

^c^Fatal.

^d^Possibly study drug related.

^e^Dose interrupted.

### Efficacy

No participants achieved a complete response (CR) or partial response (PR) (Table 4); 9/31 (29%) had stable disease as their best response. Over half (18/31 [58%]) had disease progression as their best response. Treatment duration was <3 months for 25/31 (81%) participants overall (50 mg, 3/3 [100%]; 100 mg, 12/16 [75%]; 200 mg, 10/12 [83%]). Treatment duration for 5/31 (16%) was between 3 and 6 months (100 mg, 3/16 [19%], 200 mg, 2/12 [17%]). Only 1 patient was treated for >6 months. This patient had a primary diagnosis of pleomorphic adenoma in 2009, was treated with surgery and radiotherapy between 2012 and 2015, and presented with metastatic disease in the lung and lymphatics at study entry in 2020. Although this patient achieved stable disease at the 100-mg dose level, there was a gradual increase in target lesion (lung) size over this period, and it is therefore possible that the assessment of stable disease by RECIST 1.1 was related to the natural history of their disease, rather than a slowing of disease progression following treatment with GSK3368715.

**Table 4:** Summary of best response as per RECIST 1.1 criteria.

Best response	GSK3368715 dose			
	50 mg (*n* = 3)	100 mg (*n* = 16)	200 mg (*n* = 12)	Total (*N* = 31)
Best response, *n* (%)				
Complete response	0	0	0	0
Partial response	0	0	0	0
Stable disease	0	6 (38)	3 (25)	9 (29)
Progressive disease	2 (67)	10 (63)	6 (50)	18 (58)
Not evaluable^a^	1 (33)	0	0	1 (3)
Missing	0	0	3 (25)	3 (10)
Overall response rate, *n* (%)				
Complete response + partial response	0	0	0	0
Time on GSK3368715				
Median, days (range)	53 (25, 56)	58 (13, 224)	24 (1, 114)	49 (1, 224)
<3 months, *n* (%)	3 (100)	12 (75)	10 (83)	25 (81)
3 months to 6 months, *n* (%)	0	3 (19)	2 (17)	5 (16)
>6 months to 12 months, *n* (%)	0	1 (6)	0	1 (3)
>12 months	0	0	0	0

^a^Patient did not receive study drug after the first dose.

### Pharmacokinetics

Following single and repeated oral administration, GSK3368715 was rapidly absorbed, with maximum plasma concentration (*C*_max_) reached within the first hour after dosing (Supplemental Table 3 and Supplemental Fig. 1). For 100-mg and 200-mg daily dose levels, average terminal phase half-life (*t*_1/2_) was 7.9 h and 15.9 h, respectively, following the first dose. *C*_max_ and AUC_0−24_ increased in a dose proportional manner for GSK3368715 for the dose range studied, with drug accumulation of 2.5- to 3.5-fold after repeated once-daily dosing. GSK3368715 also exhibited high PK variability (%CVb in Supplemental Table 3) with the underlying reason being unclear.

Two metabolites (GSK3963583, GSK3983164) rapidly formed with time to *C*_max_ within the first 2 h. Exposure was ~65% and 5% that of parent drug for GSK3963583 and GSK3983164, respectively. As most of the concentrations for a third metabolite, GSK3510519, were below the limit of quantification, PK analysis for this metabolite could not be performed.

### Pharmacodynamics

**Fig. 2:**
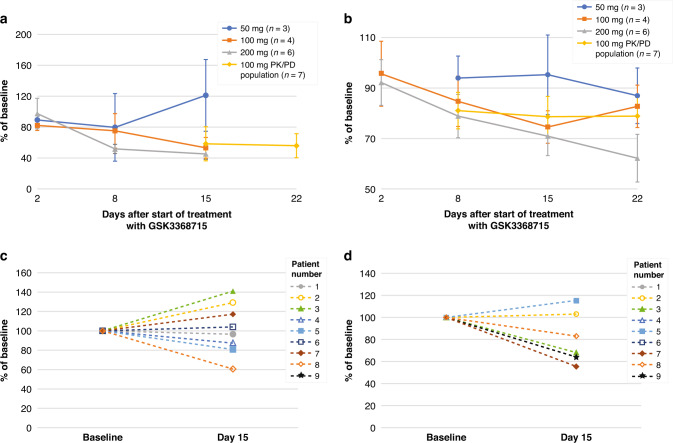
Target engagement. **a** Mean (SD) ADMA-R225-hnRNP-A1 in PBMCs in blood. **b** Mean (SD) plasma circulating free ADMA. **c** Tumor ADMA-R225-hnRNP-A1 (IHC)^a^. **d** Tumor ADMA-R225-hnRNP-A1 (LC-MS)^b^. ADMA asymmetric dimethylarginine, DE dose-escalation cohort, ICH immunohistochemistry, LC-MS liquid chromatography-mass spectrometry, PBMC peripheral blood mononuclear cells, PD pharmacodynamic, PK pharmacokinetic. ^a^IHC: formalin-fixed paraffin-embedded tumor biopsy. ^b^LC-MS: fresh frozen tumor biopsy.

Reduction of levels of ADMA-R225-hnRNP-A1 in PBMCs was time dependent with a mean (SE) reduction of 54.7% (6.92%) in the GSK3368715 200-mg dose group on day 15. A mean (SE) day 15 reduction of 43.1% (5.81%) was observed at 100 mg (Fig. 2a). Reduction in circulating free ADMA was time and dose dependent, with mean (SE) reductions on day 15 of 29.1% (7.7%) in the 200-mg dose group and 22.5% (1.98%) at 100 mg (Fig. 2b). Mean (SE) increases of 24.8% (11.9%) for cf ω-N^G^-monomethylarginine (cfMMA) and 30.2% (8.65%) for cf ω-N^G^,N’^G^-symmetric dimethylarginine (cfSDMA) were observed at 100 mg on day 15. At 200 mg, increases in cfMMA and cfSDMA were 43.8% (18.7%) and 39.8% (23.8%), respectively.

On day 15, modest and variable target engagement was observed in tumor biopsies at the 100-mg dose level, with a mean reduction (SE) of ADMA-hnRNP-A1 of 18.5% (9.6) in 4/6 patients by LC-MS and 2.08% (9.36) in 4/8 patients by IHC (Fig. 2c, d). Levels of protein SDMA were increased in formalin-fixed paired biopsies in 7/8 (88%) patients with a median (range) increase of 48.9% (−28 to 155%).

## Discussion

GSK3368715 is a first-in-class type I PRMT inhibitor that exhibited strong anticancer activity in preclinical studies. Despite this encouraging finding, and early evidence in the current study of target engagement in peripheral blood at doses of 200 mg, the study was paused due to concern over a higher-than-expected rate of TEEs and limited clinical activity as manifested by disease stability in 29% of patients. These results should be interpreted with caution, considering that only 5/31 (16%) participants had treatment for 3–6 months, and only 1 participant had treatment beyond 6 months. Taken together, the lack of observed clinical efficacy, the cumulative incidence of TEEs over a relatively short period of time, and limited and variable target engagement in the tumor at lower doses (100 mg) led to a comprehensive risk/benefit analysis and early study termination.

The expected rate of TEEs in a population of patients with advanced cancer treated in Phase I studies has been previously described [15]. Considering Khorana score [13], 1 (4%) TEE would have been expected, versus the overall observed 9 patients (29%; 95% confidence interval: 14–48%) with TEEs in this population. Three participants with TEEs had a history of prior TEEs; however, no clear trend was observed when other known risks such as tumor type and tumor burden-related conditions were considered in this population. Thus, the mechanism for the development of TEEs remains unresolved. It is notable that there was only one TEE amongst 12 patients enrolled after the amendment that introduced risk mitigation strategies for thrombosis, including exclusion of patients at high risk of TEE, initiation of treatment at the lower 100-mg dose level, consideration of prophylactic anticoagulation for those patients with a Khorana score of 2 (3/12 patients received this), and extended monitoring for TEE as DLTs. No other patients in the study received prophylactic anticoagulation, and although it is logical that this may have decreased the observed incidence of TEE after the protocol amendment, we have limited data to support this in isolation as an effective mitigation measure. Although there were no TEEs reported in preclinical models, it is not yet known whether their development could be linked to characteristics of the GSK3368715 molecule itself, or if there is an association between the mechanism of action of all type I PRMT inhibitors and the development of TEEs. Biomarkers of coagulation were not evaluated in this study but this may be useful in future studies to determine if there is a true association with TEEs and type I PRMT inhibitors before this mechanism can be effectively targeted. Further, it remains to be determined whether similar safety outcomes will be observed in studies of other drugs with similar targets (ie, drugs that inhibit protein methylation pathways or modulate epigenetic regulation). Apart from the TEEs, GSK3368715 had an otherwise manageable safety profile.

Efficacy in this unselected patient population was limited and no clear trend regarding treatment response and disease characteristics was apparent. Efficacy may have been influenced by the incidence of TEEs and low target engagement in tumor. In a subset of in vivo preclinical xenograft models, a 40–60% decrease in ADMA-hnRNP-A1 measured by IHC in tumors was associated with 80–100% tumor growth inhibition. Thus, a response would have been expected at the 200-mg dose level. While the incidence of TEEs precluded evaluation of target engagement and efficacy at 200 mg which may have confirmed a relationship between dose level and efficacy, limited tumor target engagement at 100 mg suggested an association with the lack of efficacy at this dose level. Additionally, some tumor types with frequent MTAP gene deletion were included in part 1, but MTAP loss was not required for enrollment. Only 6 patients in the study had MTAP gene deletion and of those, 4 had progressive disease with treatment exposure ranging from 15 days to 37 days and 2 achieved stable disease. The two patients who achieved stable disease included the patient with a primary diagnosis of pleomorphic adenoma who had 224 days of exposure to GSK3368715 100-mg and a patient who had a uterine perivascular epithelioid cell tumor and 57 days of treatment exposure at the 200-mg dose level. Due to the small number of patients with MTAP loss included in the study and limited exposure to treatment, no conclusions can be made regarding efficacy in this specific patient population.

Despite the findings in this study, additional investigation of PRMT inhibition remains warranted regarding, both the interplay between type I and type II PRMT (PRMT5) inhibition and the respective utility of targeting each individually for the treatment of cancer. Preclinical studies suggest a synthetic lethal relationship between PRMT5 and loss of type I PRMT function [6]. Plasma SDMA was reduced with inhibition of PRMT5 in Phase 1 studies [16–18], whereas type I PRMT inhibition usually has opposing effects on SDMA. Forced expression of MTAP in MTAP-null cell lines increased the amount of cellular SDMA in Western blots in some, but not all cell lines examined [19]. Likewise, forced expression of MTAP in MTAP-null cell lines has been shown to protect from the cytotoxic effects of type I PRMT type inhibition in some cell lines.

With respect to PRMT5, overexpression has been linked with multiple hematopoietic and solid cancers, and several selective PRMT5 inhibitors have recently been studied in Phase 1/2 trials, particularly to target tumor dependencies on PRMT5 functioning as a splicing regulator [20]. Arguably, more encouraging clinical activity in the context of a manageable safety profile has been observed with PRMT5 inhibitors versus type 1 PRMT inhibitors. For example, dose-dependent anti-proliferative activity was demonstrated with JNJ64619178 in cell lines from multiple cancer types, with pancreatic, hematological, breast, colon, lung, and ovarian cancers being the most sensitive [21]. The toxicity profile in humans was manageable, and robust target engagement with a partial response rate of 13% was observed in patients with adenoid cystic carcinoma [18]. Durable stable disease responses were achieved in other tumor types. Furthermore, evidence of clinical activity has been demonstrated for other PRMT5 inhibitors in development such as GSK3326595 and PF06939999 [16, 17]. However, no drug has yet shown sufficient activity to progress into the later stages of clinical development, and whether a PRMT5 inhibitor or type I inhibitor demonstrates favorable benefit risk in any indication to achieve a regulatory approval in the near future remains uncertain.

Overall, in the current study, which targeted type 1 PRMT in isolation, heterogeneity of the study population may be contributing to the study results.

## Conclusions

Despite promising preclinical results and observed peripheral target engagement at higher doses, the incidence of TEEs, variable target engagement at the tumor level, and observed limited clinical efficacy led to early termination of this trial. It is not known whether the lack of clinical efficacy and elevated risk of TEEs is specific to GSK3368715 or if type I PRMT inhibition may still be a viable cancer treatment alone or in combination with other therapies. No future clinical trials are planned at this time for GSK3368715 and it would be important that further development of drugs in the same class will require an understanding of the mechanism by which inhibition of type I PRMTs may impact the risk for TEEs.

## Supplementary information

Supplemental Materials

## Data Availability

Within 6 months of this publication, anonymized individual participant data, the annotated case report form, protocol, reporting and analysis plan, dataset specifications, raw dataset, analysis-ready dataset, and clinical study report will be available for research proposals approved by an independent review committee. Proposals should be submitted to www.clinicalstudydatarequest.com. A data access agreement will be required.
