# Supplementary material for: Phase 1 study of GSK3368715, a type I PRMT inhibitor, in patients with advanced solid tumors
Source: Br J Cancer. 2023 May 26;129(2):309–17. doi: 10.1038/s41416-023-02276-0 (PMC10338470; doi:10.1038/s41416-023-02276-0)
Supplement: Supplementary file 1 — Supplemental Materials [file 41416_2023_2276_MOESM1_ESM.docx]

Supplemental Materials

The following supplemental Word document includes the full inclusion and exclusion criteria for study participation (Supplemental Table 1), Khorana score predictors of venous thromboembolism (Supplemental Table 2).

derived pharmacokinetic parameters for GSK3368715 and its metabolites (Supplemental Table 3), and pharmacokinetic concentration-time plots for GSK3368715 (Supplemental Figure 1).

**Supplemental Table 1. Inclusion and exclusion criteria for study participation**

| **Inclusion Criteria** | |
| --- | --- |
| **Age** | ≥18 to years of age inclusive, at the time of informed consent |
| **Diagnosis** |  |
| Part 1 (Dose escalation, food effect, and pharmacokinetic/pharmacodynamic [PK/PD] cohorts) | - Histologically- or cytologically confirmed diagnosis of malignant solid tumor that is metastatic or nonresectable - Exhausted all standard treatment options or no longer eligible for additional standard treatment options - Evaluable disease that may be measured directly by the size of the tumor or can be evaluated by other methods - Availability of a biopsy of the tumor tissue obtained at any time from the initial diagnosis to study entry. Although a fresh biopsy obtained during screening is preferred, an archival tumor specimen is acceptable if it is not feasible to obtain a fresh biopsy. - For participants in the PK/PD cohort only, a fresh biopsy and consent for one on treatment biopsy are required for enrollment |
| Part 2 (Dose expansion) |  |
| Cohort 2A & 2B | - The availability of archival tumor tissue, or willingness to undergo a fresh biopsy to determine S-methyl-5'-thioadenosine phosphorylase (MTAP) status (any archival tumor specimen must have been obtained within 6 months prior to starting study drug unless approved by the study Medical Monitor). Local MTAP or *CDKN2A* results are acceptable for enrollment but must be confirmed through central laboratory testing. |
| Cohort 2A | - Histologically- or cytological-confirmed diagnosis of diffuse large B-cell lymphoma (DLBCL) - Relapse or refractory disease after at least 1 but not more than 4 lines of prior therapy - At least 1 measurable site of disease according to the Lugano Classification. The site of disease must be greater than 1.5 cm in the long axis regardless of short axis measurement, or greater than 1.0 cm in the short axis regardless of long axis measurement, and clearly measurable in 2 perpendicular dimensions. |
| Cohort 2B  Pancreatic cancer | - Histologically- or cytologically confirmed adenocarcinoma of the pancreas - Unresectable, locally advanced (stage III), or metastatic (stage IV) disease - Relapsed or refractory disease after at least 1 prior line of approved, systemic therapy - At least 1 measurable tumor lesion per RECIST 1.1 (Eisenhauer et al 2009) |
| Non-small cell lung cancer (NSCLC) | - Histologically- or cytologically confirmed NSCLC - Stage IV disease - Tested for presence of EML4-ALK rearrangement - Received at least 2 prior lines of approved, systemic therapy, of which 1 therapy has to be a platinum-containing regimen,   or   - Failed a first-line platinum-containing regimen in combination with an anti-PD1 monoclonal antibody and refused a second-line regimen despite being informed about the different therapeutic options and their specific clinical benefit by the investigator; the content of this informed consent discussion including the therapeutic options reviewed by the investigator needs to be documented and the participant needs to sign a specific consent form - At least 1 measurable tumor lesion per RECIST 1.1 |
| Transitional cell carcinoma of the urothelium | - Histologically- or cytologically confirmed transitional cell carcinoma (TCC) of the urothelium (urinary bladder, urethra, ureter, or renal pelvis) including mixed pathology with predominantly (ie, >50% of the histopathology sample) TCC with the exception of neuroendocrine or small-cell carcinoma - Unresectable, locally advanced (T4b) or metastatic (lymph node or visceral) disease - Relapsed or refractory disease after at least 1 prior line of approved systemic therapy - At least 1 measurable tumor lesion per RECIST 1.1 |
| **Adequate organ function as defined by:** |  |
| Hematologic |  |
| Absolute neutrophil count (ANC) | ≥1.5 x 10^9^/L |
| Hemoglobin^a^ | Solid malignancy: ≥9 g/dL  Non-Hodgkin’s Lymphoma: ≥8 g/dL |
| Platelets^a^ | ≥100 x 10^9^ /L |
| Prothrombin time (PT)/international normalized ratio (INR) and partial prothrombin time (PTT) | ≤1.5 upper limit of normal (ULN), unless participant is receiving systemic anticoagulation |
| Hepatic |  |
| Albumin | ≥2 g/dL |
| Total bilirubin | ≤1.5 x ULN  **NOTE**: Isolated bilirubin >1.5 X ULN is acceptable if:   - - Bilirubin is fractionated and direct bilirubin <35% OR - Participant has a diagnosis of Gilbert’s syndrome |
| Alanine aminotransferase (ALT) | Part 1 and 2: ≤2.5 x ULN  Part 2 ONLY: <5 x ULN is acceptable for participants with  documented liver metastases/tumor infiltration |
| Renal |  |
| Calculated creatinine clearance by  Chronic Kidney Disease Epidemiology  Collaboration (CKD-EPI) equation or  measured from 24h urine | ≥50 mL/min |
| Cardiac |  |
| Ejection fraction | ≥Lower limit of normal (LLN) by ECG (minimum of 50%)/multigated acquisition scan (MUGA) |
| Echocardiogram (ECG): QT corrected for heart rate by Fridericia’s formula (QTcF)^b^ | <450 ms |
| **Eastern Cooperative Oncology Group (ECOG) performance status of 0 or 1** |  |
| **Ability to swallow and retain orally administered medication** |  |
| **Sex** |  |
| Female participants | A female participant is eligible to participate if she is not pregnant or breastfeeding, and at least one of the following conditions applies:   - Is not a woman of childbearing potential (WOCBP)   OR   - Is a WOCBP and using a contraceptive method that is highly effective, with a failure rate of <1%, during the intervention period and for at least 120 days, corresponding to the time needed to eliminate any study intervention(s) (eg, 5 terminal half-lives) after the last dose of study intervention. The investigator should evaluate the effectiveness of the contraceptive method in relationship to the first dose of study intervention.   A WOCBP must have a negative highly sensitive pregnancy test (urine as  required by local regulations) within 7 days before the first dose of study  intervention  The investigator is responsible for review of medical history, menstrual history,  and recent sexual activity to decrease the risk for inclusion of a woman with an  early undetected pregnancy |
| Male participants | Male participants are eligible to participate if they agree to the following during the intervention period and for at least 100 days, corresponding to time needed to eliminate study intervention(s) (eg, 5 terminal half-lives) plus 90 days after  the last dose of study intervention:   - Refrain from donating sperm   Plus, either:   - Be abstinent from heterosexual or homosexual intercourse as their preferred and   usual lifestyle (abstinent on a long term and persistent basis) and agree to remain  abstinent  OR   - Must agree to use contraception/barrier as detailed below:   - Agree to use a male condom and should also be advised of the benefit   for a female partner to use a highly effective method of contraception  as a condom may break or leak when having sexual intercourse with a  WOCBP who is not currently pregnant   - - Agree to use male condom when engaging in any activity that allows   for passage of ejaculate to another person |
| **Informed consent** | Capable of giving signed informed consent which includes compliance with the requirements and restrictions listed in the informed consent form (ICF) and in the protocol |
| **Exclusion criteria** | |
| **Prior or concomitant diseases** | - History of malignancy other than the disease under study   **EXCEPT:** Participants who have been disease free for 5 years, or  participants with a history of completely resected nonmelanoma skin cancer or  successfully treated in situ carcinoma are eligible. Participants with second  malignancies that are indolent or definitively treated may be enrolled even if less  than 5 years have elapsed since treatment. Consult GSK Medical Monitor if  unsure whether second malignancies meet requirements specified above   - Primary central nervous system (CNS) tumors, glioblastoma multiforme (GBM),   symptomatic or untreated leptomeningeal or brain metastases or spinal cord  compression  **EXCEPT**: Participants previously treated for these conditions that  have had stable CNS disease (verified with consecutive imaging studies) for  >1 month, are asymptomatic and off corticosteroids, or are on a stable dose of  corticosteroids for at least 1 month prior to study day 1 are permitted. Stability of  brain metastases must be confirmed with imaging. Participant treated with gamma  knife therapy can be enrolled 2 weeks postprocedure as long as there are no postprocedure  complications/they are stable   - Any severe or uncontrolled systemic diseases (eg, unstable or uncompensated   respiratory, hepatic, renal, cardiac disease, or clinically significant bleeding episodes,  or active infection)   - Any serious and/or unstable preexisting medical or psychiatric disorder, or other   conditions that could interfere with participant’s safety, obtaining informed consent,  or compliance to the study procedures, in the opinion of the investigator   - Any clinically significant gastrointestinal (GI) abnormalities that may alter absorption such as malabsorption syndrome or major resection of the stomach and/or bowels - History of known human immunodeficiency virus (HIV) infection or positive HIV test result at screening - Presence of hepatitis B surface antigen (HBsAg) or positive hepatitis C antibody test result at screening to first dose of study intervention   **NOTE:** Participants with positive hepatitis C antibody due to prior resolved disease can be enrolled only if a confirmatory negative hepatitis C RNA polymerase chain reaction (PCR) is obtained   - Any of the following cardiac abnormalities:   a) Uncontrolled high blood pressure  b) Any history of coronary artery disease, including acute coronary syndromes,  myocardial infarction, unstable angina, and history of coronary angioplasty,  or stenting  c) Presence of a cardiac pacemaker or implanted defibrillator.  d) AV-block (asymptomatic 2nd degree type II or 3rd degree and any degree AV  block if related to heart disease or if symptomatic), RBBB (right bundle  branch block), LBBB (left bundle branch block), and any fascicular  hemiblocks  e) A QRS interval at Screening or Baseline ≥110 msec  f) Patients with any symptomatic or sustained arrhythmias (past or present),  including but not limited to:   - Atrial fibrillation - Atrial flutter - Ventricular tachycardia - Ventricular fibrillation - Supraventricular tachycardia   g) Current or past congestive heart failure  h) Evidence of a left ventricular ejection fraction below the institutional lower  limit of normal on screening ECG  i) Evidence of significant structural heart disease on echocardiography at  Screening (including any valvular disease greater than “mild” in severity)  j) Cardiac troponin > upper limit of the reference range at screening |
| **Prior or concomitant anticancer therapies** | - Treatment with any local or systemic antineoplastic therapy or investigational anticancer agent within 14 days or 4 half-lives, whichever is longer, up to a maximum wash-out period of 28 days prior to initiation of study drug administration   **EXCEPT:** Antiandrogen therapies for prostate cancer, such as bicalutamide, must be stopped 28 days prior to first dose of GSK3368715. Second-line hormone therapies such as enzalutamide or abiraterone should be stopped 14 days prior to enrollment. Participants with prostate cancer may remain on (i) luteinizing hormone-releasing hormone (LHRH) agonists or antagonists and/or (ii) low-dose prednisone or prednisolone (up to 10 mg/day)   - Nitrosureas and mitomycin C must be stopped within 42 days prior to first dose of GSK3368715 - Allogeneic hematopoietic stem cell transplantation - Toxicities from previous anticancer therapies have not resolved to baseline or NCI common terminology criteria for adverse events (CTCAE) v5 ≤grade 1 (except fatigue and alopecia [permissible at any grade] and peripheral neuropathy [which must be ≤grade 2]) at the time of starting study intervention |
| **Prior or concomitant therapies** | - Major surgery (ie, requiring general anesthesia) within 3 weeks before screening, or not fully recovered from major surgery, or major surgery planned during study participation   **EXCEPT:** Planned surgical procedures to be conducted under local anesthesia are allowed   - Prior organ transplantation - Current use of a prohibited medication or planned use of any forbidden medications during intervention with GSK3368715 - History of sensitivity to any of the study medications, or components thereof, or a history of drug or other allergy that, in the opinion of the investigator or Medical Monitor, contraindicates their participation - Participant is considered high risk for venous thromboembolism (VTE) as defined by either Khorana Score of ≥3, or prior medical history of VTE   **NOTE:** Participants with Khorana score of 2 should be considered for prophylactic anticoagulation per current ASCO guidelines (Key et al 2019) if deemed appropriate by the investigator |

^a^ Participants that require transfusion or initiation of growth factor support in order to achieve

necessary platelet and/or hemoglobin must maintain adequate values for at least 7 days without

transfusion or while on growth factor in order to be eligible for participation

^b^ Baseline QTc interval using Fridericia’s formula based on average of triplicate ECGs obtained over a brief recording period.

**References**

1. Eisenhauer EA, Therasse P, Bogaerts J, et al. New response evaluation criteria in solid tumors: Revised RECIST guidelines (version 1.1). Eur J Cancer. 2009;45:228-247.
2. Key NS, Khorana AA, Kuderer NM, et al. Venous Thromboembolism Prophylaxis and

Treatment in Patients With Cancer: ASCO Clinical Practice Guideline Update. J Clin Oncol. 2019;38(5):496-520.

Supplemental Table 2. Khorana Score Predictors of Venous Thromboembolism^1^

| Risk factor | Score |
| --- | --- |
| Stomach or pancreatic cancer | 2 |
| Lung, lymphoma, gynecologic, bladder or testicular cancer | 1 |
| Prechemotherapy platelet count ≥350 x 10^9^/L | 1 |
| Hemoglobin level <100 g/L or use of red cell growth factors | 1 |
| Prechemotherapy leukocyte count >11 x 10^9^/L | 1 |
| BMI ≥35 kg/m^2^ | 1 |

BMI, body mass index.

**References**

1. Khorana AA, Kuderer NM, Culakova E, Lyman GH, Francis CW. Development and validation of a predictive model for chemotherapy-associated thrombosis. *Blood*. 2008;111(10):4902–7. doi: 10.1182/blood-2007-10-116327

Supplemental Table 3. Summary of Derived Pharmacokinetic Parameters (PK/PD cohort)

|  | **50-mg dose escalation cohort (N=3)** | | **100-mg dose escalation cohort (N=6)** | | **100-mg PK/PD cohort**  **(N=10)** | | **100-mg total**  **(N=16)** | | **200 mg**  **(N=12)** | |
| --- | --- | --- | --- | --- | --- | --- | --- | --- | --- | --- |
| **Parameter** | **Day 1** | **Day 15** | **Day 1** | **Day 15** | **Day 1** | **Day 15** | **Day 1** | **Day 15** | **Day 1** | **Day 15** |
| **GSK3368715** |  |  |  |  |  |  |  |  |  |  |
| **AUC_0-24_ (h•ng/mL)** | **(n=1)** | **(n=2)** | **(n=3)** | **(n=5)** | **(n=4)** | **(n=9)** | **(n=7)** | **(n=14)** | **(n=11)** | **(n=9)** |
| Geometric mean (%CVb) | 259.5 (--) | 581.9 (101.4) | 1020.7 (26.7) | 1479.6 (79.7) | 937.6 (30.9) | 1628.8 (60.4) | 972.3 (27.1) | 1573.8 (64.2) | 1274.7 (43.8) | 4861.4 (45.3) |
| **C_max_ (ng/mL)** | **(n=3)** | **(n=3)** | **(n=6)** | **(n=5)** | **(n=10)** | **(n=9)** | **(n=16)** | **(n=14)** | **(n=12)** | **(n=9)** |
| Geometric mean (%CVb) | 107.2 (48.8) | 111.9 (48.5) | 145.0 (207.3) | 362.9 (115.9) | 237.6 (62.3) | 382.7 (67.4) | 197.4 (112.1) | 375.5 (79.9) | 336.8 (72.6) | 729.2 (42.8) |
| **t_max_ (h)** | **(n=3)** | **(n=3)** | **(n=6)** | **(n=5)** | **(n=10)** | **(n=9)** | **(n=16)** | **(n=14)** | **(n=12)** | **(n=9)** |
| Median (range) | 0.28 (0.25, 1.1) | 3.0 (0.5, 3.1) | 0.5 (0.3, 3.1) | 0.9 (0.5, 1.1) | 0.5 (0.3, 3.1) | 1.0 (0.5, 2.1) | 0.5 (0.3, 3.1) | 0.9 (0.5, 2.1) | 1.1 (0.4, 3.1) | 1.0 (0.5, 3.1) |
| **t_1/2_ (h)** | **(n=0)** | **(n=0)** | **(n=1)** | **(n=1)** | **(n=1)** | **(n=1)** | **(n=2)** | **(n=2)** | **(n=4)** | **(n=4)** |
| Geometric mean (%CVb) | -- | -- | 7.2 (--) | 1.8 (--) | 8.8 (--) | 1.4 (--) | 7.9 (12.6) | 1.6 (15.6) | 15.9 (71.5) | 2.9 (43.5) |
| **GSK3963583** |  |  |  |  |  |  |  |  |  |  |
| **AUC_0-24_ (h•ng/mL)** | **(n=0)** | **(n=1)** | **(n=1)** | **(n=5)** | **(n=3)** | **(n=9)** | **(n=4)** | **(n=14)** | **(n=8)** | **(n=8)** |
| Geometric mean (%CVb) | -- | 841.7 (--) | 737.0 (--) | 769.3 (53.7) | 620.6 (22.4) | 815.4 (32.8) | 647.9 (657.6) | 798.7 (39.0) | 773.2 (45.0) | 1407.7 (29.2) |
| **C_max_ (ng/mL)** | **(n=3)** | **(n=3)** | **(n=6)** | **(n=5)** | **(n=10)** | **(n=9)** | **(n=16)** | **(n=14)** | **(n=11)** | **(n=9)** |
| Geometric mean (%CVb) | 22.7 (82.0) | 28.7 (68.6) | 50.6 (134.5) | 91.9 (76.6) | 91.3 (48.0) | 86.3 (24.0) | 73.1 (86.3) | 88.3 (44.1) | 126.3 (69.0) | 85.9 (99.2) |
| **t_max_ (h)** | **(n=3)** | **(n=3)** | **(n=6)** | **(n=5)** | **(n=10)** | **(n=9)** | **(n=16)** | **(n=14)** | **(n=11)** | **(n=9)** |
| Median (Range) | 1.5 (0.5, 3.1) | 4.1 (1.0, 5.8) | 1.6 (1.0, 4.0) | 1.0 (0.5, 3.0) | 1.1 (0.3, 3.1) | 1.1 (0.5, 3.1) | 1.1 (0.3, 4.0) | 1.1 (0.3, 3.1) | 2.0 (0.9, 4.3) | 3.1 (1.0, 4.1) |
| **t_1/2_ (h)** | **(n=0)** | **(n=0)** | **(n=0)** | **(n=0)** | **(n=0)** | **(n=0)** | **(n=0)** | **(n=0)** | **(n=3)** | **(n=3)** |
| Geometric mean (%CVb) | -- | -- | -- | -- | -- | -- | -- | -- | 9.2 (38.9) | 1.0 (44.5) |
| **GSK3983164** |  | - |  |  | -- |  |  |  |  |  |
| **AUC_0-24_ (h•ng/mL)** | -- | -- | **--** | **(n=1)** | **--** | **(n=3)** | -- | **(n=4)** | -- | **(n=8)** |
| Geometric mean (%CVb) | -- | -- | -- | 335.0 (--) | -- | 230.8 (21.1) | -- | 253.3 (25.6) | -- | 362.1 (31.0) |
| **C_max_ (ng/mL)** | --- | - | **(n=4)** | **(n=5)** | **(n=9)** | **(n=9)** | **(n=13)** | **(n=14)** | **(n=11)** | **(n=9)** |
| Geometric mean (%CVb) | -- | -- | 10.3 (59.4) | 15.1 (89.6) | 10.2 (59.4) | 15.3 (29.5) | 10.1 (58.4) | 15.2 (51.2) | 16.8 (77.7) | 28.0 (55.4) |
| **t_max_ (h)** | --- | -- | **(n=4)** | **(n=5)** | **(n=9)** | **(n=9)** | **(n=13)** | **(n=14)** | **(n=11)** | **(n=9)** |
| Median (range) | -- | -- | 2.1 (1.0, 4.0) | 1.0 (0.5, 3.0) | 1.1 (0.3, 3.1) | 1.1 (0.5, 3.1) | 1.1 (0.3, 4.0) | 1.1 (0.5, 3.1) | 3.0 (0.9, 4.3) | 3.1 (1.0, 4.1) |
| **t_1/2_ (h)** | -- | -- | -- | -- | --- | -- | -- | -- | -- | -- |

PD, pharmacodynamic; pharmacokinetic.

**Supplemental Figure 1. Median Concentration-Time Profile on Day 1 and Day 15 Following Administration of GSK3368715 100 mg (Dose Escalation and PK/PD Populations)**


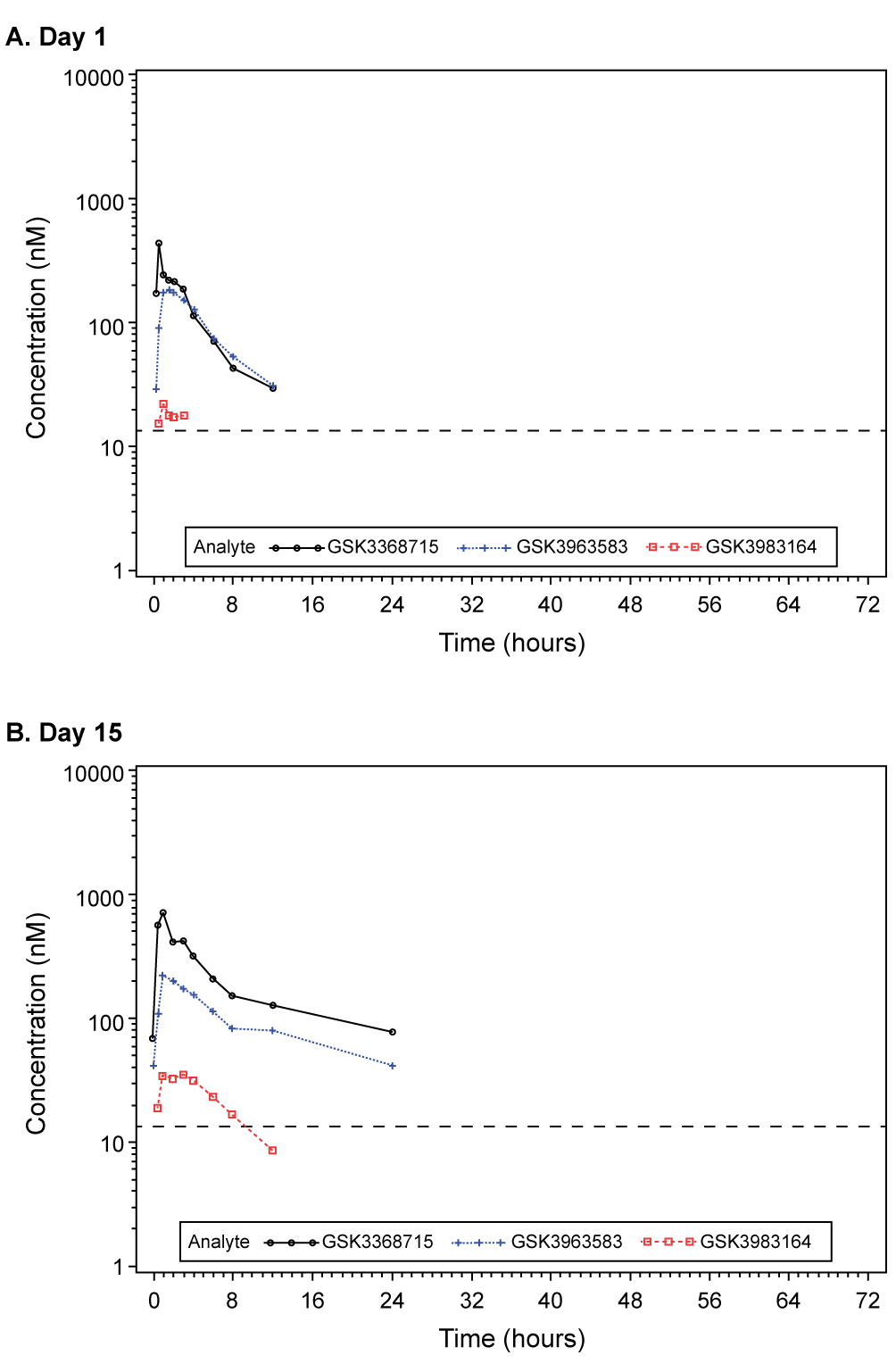


Dashed lines indicate the Lower Limit of Quantification (LLQ).
